# *Baylisascaris procyonis* on the rise in Europe: a comprehensive review and analysis of occurrence data

**DOI:** 10.1007/s00436-025-08611-z

**Published:** 2025-12-09

**Authors:** Anne Steinhoff, Robin Stutz, Anna Viktoria Schantz, Norbert Peter, Dorian D. Dörge, Sven Klimpel

**Affiliations:** 1 Institute for Ecology, Evolution and Diversity, Goethe-University, Max-von-Laue-Str. 13, Frankfurt/Main, Germany; 2 Senckenberg Biodiversity and Climate Research Centre, Senckenberg Gesellschaft für Naturforschung, Senckenberganlage 25, Frankfurt/Main, Germany

**Keywords:** *Baylisascaris procyonis*, Raccoon, Introduced species, Europe, Zoonosis

## Abstract

The raccoon roundworm (*Baylisascaris procyonis*) was introduced to Europe by its final host, the North American raccoon (*Procyon lotor*), which has been spreading uncontrollably there since the early 20th century. The raccoon is gaining importance due to the spread of the raccoon roundworm, which is pathogenic to humans and can cause severe disease through oral ingestion of infectious egg stages. Totally, 146 raccoons from central and western Germany were examined for *B. procyonis* via necropsy. In addition, an extensive literature search was conducted on the occurrence of *B. procyonis* in Europe. Of the 146 raccoons examined in this study, 66.4% were infected with *B. procyonis*: 77.4% in Hesse (*n* = 84), 51.1% in Thuringia (*n* = 45), and 52.9% in North Rhine-Westphalia (*n* = 17). The literature review revealed the presence of *B. procyonis* in wild raccoons in nine European countries. In three additional countries, infections were detected in raccoons or other animal species in captivity. The studies show a steady expansion of the distribution area, which is probably underestimated due to insufficient or absent data collection. Five animal species have been identified as paratenic/accidental hosts in Europe, with all infections documented in zoos/animal husbandry. There is a lack of data on paratenic hosts in the wild in Europe. There are three documented cases of Baylisascariasis in Europe. The results of the present study highlight the need for further research into *B. procyonis* in Europe, particularly in relation to ongoing urbanization and growing raccoon populations.

**Graphical Abstract:**

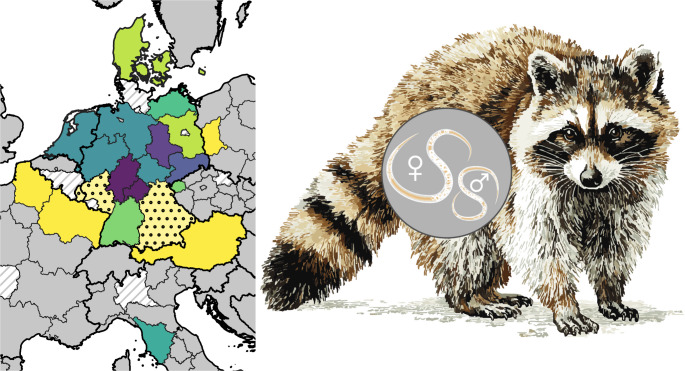

**Supplementary Information:**

The online version contains supplementary material available at 10.1007/s00436-025-08611-z.

## Introduction

Since its release and escape from fur farms in the early 20th century, the North American raccoon (*Procyon lotor*) has spread throughout Central Europe, with its populations continuing to grow uncontrollably (Cunze et al. 2023, 2025). The raccoon has now established itself in several European countries (Aliev and Sanderson 1966; Lutz 1984; Kauhala 1996; García et al. 2012; Salgado 2018; Schally et al. 2024). There is evidence of various founder populations in Central Europe. In addition to the known dispersal points, this indicates further release events, which presumably originated from private sources (Alda et al. 2013; Biedrzycka et al. 2014; Fischer et al. 2015, 2017; Larroque et al. 2023; Garofalo et al. 2024).

The raccoon is considered an invasive species of Union concern (Regulation (EU) No. 1143/2014) and, in addition to its negative impact on local ecosystems, plays a decisive role in the spread of pathogens (Keller et al. 2022; Peter et al. 2023, 2024). To study and monitor the spread of raccoon populations in Europe, their ecological impact and associated zoonoses, national and international projects have been established in recent years, such as the ZOWIAC research project and the open, collaborative EURORACCOON research project. As raccoon populations continue to spread into urban areas, they are becoming epidemiologically relevant to humans. One pathogen that has been insufficiently researched in Europe is the raccoon roundworm *Baylisascaris procyonis*. The raccoon roundworm is a human pathogenic parasite that can cause larva migrans in humans (Kazacos 2001). The disease is characterized by three clinical presentations in which larvae migrate through specific body tissues and organs: ocular larva migrans (OLM), visceral larva migrans (VLM), and neural larva migrans (NLM (Gavin et al. 2005). Humans become infected by accidentally ingesting infectious eggs found in soil, water, or on objects contaminated with raccoon feces (Kazacos 2001, 2016). If the larvae migrate through the body and into organs (larva migrans), the infection can have serious consequences (Graeff-Teixeira et al. 2016). While most infections appear to be asymptomatic, there have been several confirmed cases of Baylisascariasis in North America, most of which resulted in permanent neurological damage or death (Kazacos 2016; Weese and Stull 2025). In addition, it is assumed that many cases remain undetected or are misdiagnosed due to the nonspecific symptoms (Sorvillo et al. 2002; Kazacos 2016). In Europe, the diagnosis of Baylisascariasis in humans is further complicated by the lack of widely available diagnostic tests. A definitive diagnosis using Western blot assay is currently only possible at the CDC in the US/Canada (Rascoe et al. 2013; Graeff-Teixeira et al. 2016).

Adult *B. procyonis* parasitize the small intestine of the North American raccoon, which is the definitive host (Fig. 4). Female nematodes excrete 115,000 to 179,000 eggs per day, which are released into the environment via the raccoons’ feces (Kazacos 2001). One gram of feces contains an average of 16,000 to 26,000 eggs (Kazacos 2001; Reed et al. 2012). In the environment, at adequate temperature and humidity, the eggs develop into infectious L2 larvae within 11–14 days, which can survive for several years without losing their infectivity (Kazacos 2001; Stutz et al. 2024). L_2_- larvae in eggs can be ingested directly by juvenile raccoons and lead to infection, while infection of adult raccoons usually occurs through the ingestion of L_3_-larvae from the tissue of paratenic hosts (Kazacos 2001). The paratenic hosts ingest the infectious eggs containing the L_2_-larvae from the environment, which then migrate to various organs (Graeff-Teixeira et al. 2016). A small proportion of the larvae migrate to the central nervous system (CNS) and cause damage, leading to weakening or death of the paratenic host (Gavin et al. 2005). Infected paratenic hosts are ingested by adult raccoons, where the larvae develop into adult nematodes (Kazacos 2001). More than 150 species (primarily birds and mammals) are known to serve as paratenic hosts (Graeff-Teixeira et al. 2016).

Germany is considered to be the main area of distribution for both raccoons and the raccoon roundworm in Europe, where both species have been established for decades (Heddergott 2020). Considering the risk of infection for humans, the fact that there are currently no standardized and reliable testing methods for Baylisascariasis in Europe, and the continuous spread of raccoons, it is necessary to investigate the occurrence and intensity of *B. procyonis* infections in European raccoon populations. In order to provide an overview of the current distribution of *B. procyonis* in Europe and to identify possible research needs, new data from Germany were collected in the present study and evaluated in conjunction with an extensive literature review.

## Methods

### Raccoon sampling and data collection

A total of 146 raccoons were tested for infection with *B. procyonis*. All animals were sampled between March 2022 and November 2024 in the context of legal hunting. Metadata on the time and place of capture were recorded. A total of 84 (57.5%) raccoons came from Hesse (Wetterau district (3), Main-Kinzig district (32), Wiesbaden (49)), 45 (30.8%) from Thuringia (Wartburg district (35), Sömmerda (10)), and 17 (11.6%) from North Rhine-Westphalia (Aachen district). The animals were stored at −20 °C and thawed over a period of 24 h prior to examination. The sex and total weight of the raccoons were documented. The raccoons were dissected according to Peter et al. (2023). The gastrointestinal tract was examined for the presence of *B. procyonis*, and specimens were transferred to 70% ethanol for morhological identification. Species identification was based on morphological characteristics according to Sprent (1968). Parasitological parameters (prevalence P [%], mean intensity mI, intensity I, mean abundance mA) were calculated according to Klimpel et al. (2019).

### Literature research and data analysis

An extensive literature search was conducted to compile data on the occurrence of *B. procyonis* in Europe. All publications that were publicly available in accessible media (e.g., Google Scholar, PubMed) were used for the analysis. In order to identify further relevant publications, the reference lists of the publications included in the full-text screenings were searched manually. Only peer-reviewed publications presenting new, independently collected data from Europe were included in the analysis. The spread of the raccoon roundworm was considered at NUTS levels (Nomenclature of Territorial Units for Statistics) for each country. All research data on *B. procyonis* in raccoons in Europe were compiled in a summary table (Table S1) and provided with the corresponding literature references (Stefanski and Zarnowski 1951; Sprehn and Haakh 1956; Roth 1968; Tscherner 1974; Tenora and Staněk 1990; Bauer et al. 1992; Lux and Priemer 1995a, b; Gey 1998; Hohmann et al. 2002; Winter et al. 2005; Brinch 2006; Brandes 2007; Bartoszewicz et al. 2008; Helbig 2011; Popiołek et al. 2011; Anheyer-Behmenburg 2013; Davidson et al. 2013; Karamon et al. 2014; Al-Sabi et al. 2015; Jimenez Martinez et al. 2015; Rentería-Solís 2015; Schwarz et al. 2015; Duscher et al. 2017, 2021; Michler 2017; Osten-Sacken et al. 2018; Rentería-Solís et al. 2018, 2020, 2025; Biedrzycka et al. 2020b; Heddergott et al. 2020, 2023; Romeo et al. 2021; Lombardo et al. 2022, 2023; Maas et al. 2022; Sanjuán et al. 2022; Peter et al. 2023, 2024; Reinhardt et al. 2023; Frantz et al. 2024; House 2024; Umhang et al. 2024; Benovics et al. 2025). To visualize the spread of *B. procyonis*, the prevalence rates were sorted by region and study period. The division into the time periods 1990–2004 and 2005–2025 was based on the available prevalence data and the expansion history of the raccoon. In 2005, the raccoon was in the early stages of expansion in Central Europe (Salgado 2018).

## Results

### *Baylisascaris procyonis*- infestation of raccoons examined in this study

In 97 of the 146 ( = 66.4%) examined raccoons, an infestation with *B. procyonis* was detected, with the highest prevalence found in the sample from Sömmerda in Thuringia with = 80.0%, followed by Wiesbaden (P = 79.6%) and the Main-Kinzig district (P = 75.0%) in Hesse (Table 1). The lowest prevalence (P = 42.9%) was detected in the Wartburg district in Thuringia. The mean intensities ranged between mI = 11.0 (Wartburg district) and mI = 39.1 (Aachen district). The maximum intensity was determined to be maxI = 204 nematodes in one animal from Wiesbaden. The mean abundance was highest in the Aachen district (mA = 20.7), followed by Sömmerda (mA = 20.5) with the lowest in the Wartburg district (mA = 4.7).

**Table 1:** Parasitological calculations for the infestation of the raccoons examined with *Baylisascaris procyonis* by district: prevalence (P [%]), intensity (mI), minimum intensity (minI), maximum intensity (maxI) and mean abundance (mA)

Area	n	P [%]	mI	minI	maxI	mA
**Hesse**	**84**	**77.4**	**15.2**	**1**	**204**	**11.7**
Main-Kinzig district	32	75.0	11.3	1	78	8.5
Wetterau district	3	66.7	24.0	11	37	16.0
Wiesbaden	49	79.6	17.1	1	204	13.6
**Thuringia**	**45**	**51.1**	**16.1**	**1**	**55**	**8.2**
Sömmerda	10	80.0	25.6	2	55	20.5
Wartburg district	35	42.9	11.0	1	46	4.7
**North Rhine-Westphalia** (Aachen district)	**17**	**52.9**	**39.1**	**1**	**132**	**20.7**
**Total**	**146**	**66.4**	**17.6**	**1**	**204**	**11.7**

## Occurrence of the raccoon roundworm in Europe

### In raccoons

A total of 45 publications between 1951 and 2025 documented positive or negative findings of *B. procyonis* infections in raccoons in Europe; these are listed in the appendix table (Table S1). The compilation of the literature resulted in evidence of *B. procyonis* in captive raccoons and wild animals in 10 European countries. In 48.9% of the publications, the evidence was obtained through necropsy, in which adult and juvenile nematodes were removed from raccoons by dissection of the gastrointestinal tract and identified morphologically and/or genetically at the species level (Gey 1998; Peter et al. 2023). In 20% of the publications, evidece was provided by fecal analysis, in which *B. procyonis* eggs were extracted from raccoon feces using flotation methods and morphologically identified or their presence was detected trough PCR and sequencing (Gey 1998; Bartoszewicz et al. 2008; Rentería-Solís et al. 2025). In 2.3% of publications, detection was achieved through treatment with anthelmintics, in which adut and juvenile nematodes in freshly deposited raccoon feces were visualized and identified morphologically and/or genetically at the species level (Maas et al. 2022; Frantz et al. 2024). In 26.7% of the publications, detection was based on a combination of necropsies and fecal analyses and/or treatment; in one publication, the method used to examine positive raccoons was not specified (Table S1).

The oldest evidence of a raccoon infected with *B. procyonis* was found at Lodz Zoo (Poland) in 1948. This was also the first time that *B. procyonis* was described in detail (Stefanski and Zarnowski 1951) So far, evidence of *B. procyonis* infections in captive raccoons has been found in six countries (Stefanski and Zarnowski 1951 in Poland; Sprehn and Haakh (1956), Roth (1968), Tscherner (1974) and Brandes (2007) in Germany; Tenora and Staněk (1990) in the Czech Republic; Brinch (2006) and Al-Sabi et al. (2015) in Denmark; Davidson et al. (2013) in Norway; Jimenez Martinez et al. (2015) in Spain). In Norway, there is only evidence of infection in captive raccoons, not in wild ones (Davidson et al. 2013).

**Fig. 1:**
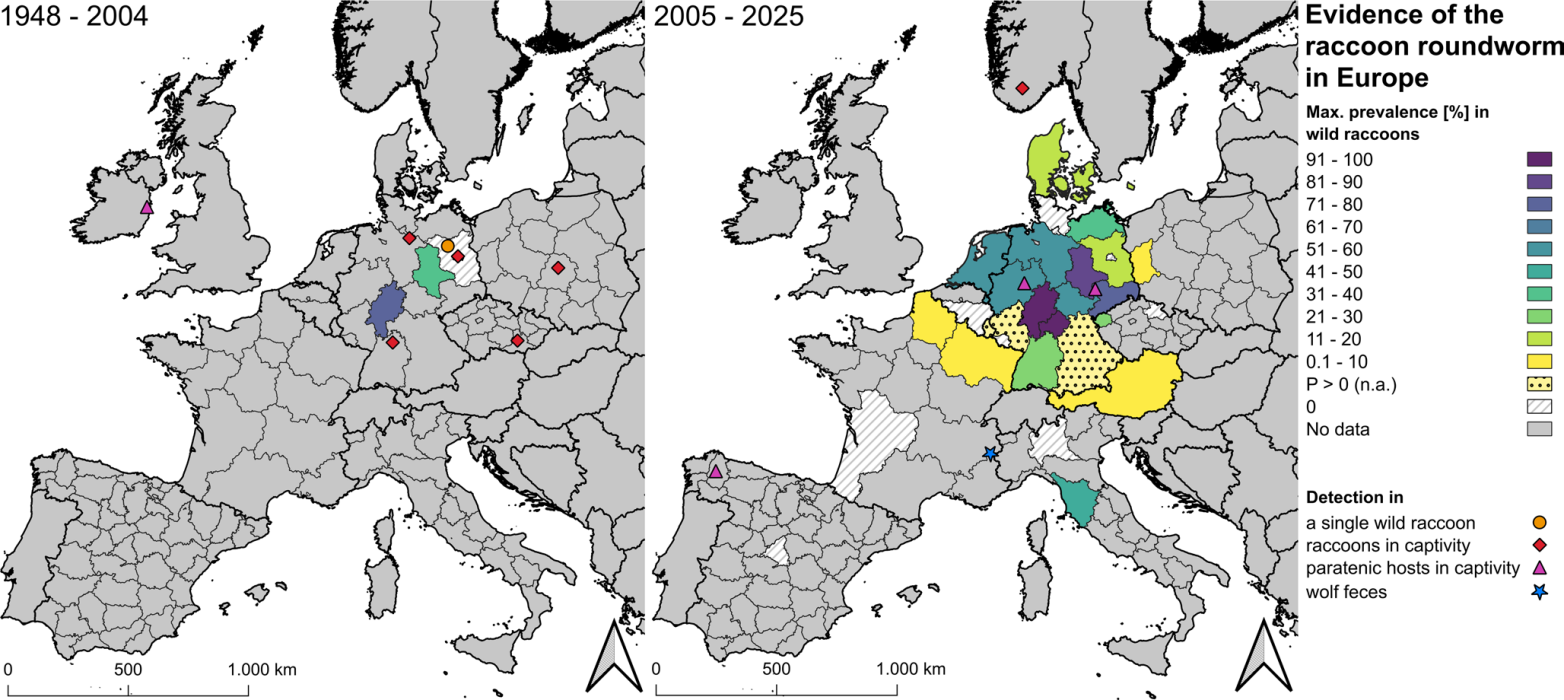
Maximum prevalence in free ranging raccoons and individual records of *Baylisascaris procyonis* in Europe at NUTS level 0, 1 or 2 for the time periods 1948–2004 (left) and 2005–2025 (right). Prevalence data is based on Table 2. n.a. = not available

In total, *B. procyonis* infections have been detected in wild raccoons in nine countries (e.g. Austria (Duscher et al. 2021); Czech Republic (Benovics et al. 2025); Denmark (Duscher et al. 2021); France (Umhang et al. 2024); Germany (Heddergott et al. 2020); Italy (Lombardo et al. 2022); Luxembourg (Frantz et al. 2024); the Netherlands (Maas et al. 2022); Poland (Bartoszewicz et al. 2008)). The first record was made in the early 1990 s in Germany (Hesse) (Bauer et al. 1992). The documented prevalences are listed in Table 2. An overview of the distribution, individual references and maximum prevalences before and after 2005 is shown in Fig. 1, based on Table 3 and S1. For regions without available prevalence data, qualitative evidence according to Frantz et al. (2024) and Heddergott et al. (2020) was indicated (“P > 0 (n.a.)” or 0%).

**Table 2:** Prevalences (P [%]) of *Baylisascaris procyonis* in free ranging raccoons in Europe

Country	Region	Investigation periods [years]	n	P [%]	Study Method	References
Austria	/	2010–2016	8	0	N	Duscher et al. (2017)
		2017–2019	42	2.4	N	Duscher et al. (2021)
Belgium	Walloon Region	2012–2015	50	0	N	Maas et al. (2022)
Czech Republic	Hradec Kralove Region	2012–2017	62	0	N	Biedrzycka et al. (2020)
	Karlovy Vary Region	2023–2025	10	30	N	Benovics et al. (2025)
Denmark	/	2009–2015	18	11.1	N	Al-Sabi et al. (2015)
France	Hauts-de-France & Grand-Est	2011–2021	208	0.5	N	Umhang et al. (2024)
	Nouvelle-Aquitaine	2019–2022	92	0	N	Umhang et al. (2024)
Germany	/	2008–2018	8,184	43.6	N	Heddergott et al. (2020)
	Baden-Württemberg	2019–2020	101	28.7	N	Reinhardt et al. (2023)
	Bavaria (Lower Franconia)	2017–2021	27	96.3	N	Peter et al. (2023)
	Berlin	2006–2013	987	0	N	Rentería-Solís (2015)
	Brandenburg	1993	7	0	N	Lux and Priemer (1995a)
		1993–1995	41	0	N	Lux and Priemer (1995b)
		2008–2013	762	0	N	Schwarz et al. (2015)
		2020–2022	36	19.4	N	Peter et al. (2024)
	Hesse	n.a.	121	72	N	Bauer et al. (1992)
		1990–1992	147	71.4	N	Gey (1998)
		1999	15	80	F	Hohmann et al. (2002)
		2019–2020	22	50	N + F	Rentería-Solís et al. (2025)
		2017–2021	207	94.7	N	Peter et al. (2023)
		2020–2022	36	91.7	N	Peter et al. (2024)
		2022–2024	84	77.4	N	New data
	Lower Saxony	2011–2013	457	51.4	N	Anheyer-Behmenburg (2013)
		2019–2020	38	44.7	N + F	Rentería-Solís et al. (2025)
	Mecklenburg-Western Pomerania	2006–2011	400	0	F	Michler (2017)
		2006–2013	100	0	N	Rentería-Solís (2015)
		2019–2020	13	30.8	N + F	Rentería-Solís et al. (2025)
	North Rhine-Westphalia	2019–2020	16	31.2	N + F	Rentería-Solís et al. (2025)
		2022–2024	17	52.9	N	New data
	Saxony	2017–2018	32	75	N	Rentería-Solís et al. (2018)
		2019–2020	53	39.6	N + F	Rentería-Solís et al. (2025)
	Saxony-Anhalt	2002–2004	56	39.3	N	Winter et al. (2005)
		2012	6	16.7	N	Schwarz et al. (2015)
		n.a.	47	44.7	F	Helbig (2011)
		2016–2017	197	32.5	N	House (2024)
		2019–2020	12	66.7	N + F	Rentería-Solís et al. (2025)
		2020–2021	181	48.6	N	Heddergott et al. (2023)
		2020–2022	36	88.9	N	Peter et al. (2024)
	Thuringia	2019–2020	5	0	N + F	Rentería-Solís et al. (2025)
		2022–2024	45	51.1	N	New data
Italy	Lombardy	2017–2019	67	0	N	Romeo et al. (2021)
	Tuscany	2021	21	33.3	N	Lombardo et al. (2022)
		2020–2022	62	41.9	N	Lombardo et al. (2023)
Luxembourg	/	2008–2018	26	0	N	Heddergott et al. (2020)
Netherlands	/	2015–2020	29	58.6	N + F + T	Maas et al. (2022)
Poland	Lubusz Voivodeship	2005–2007	27	3.7	F	Bartoszewicz et al. (2008)
		2006–2007	91	3.3	F	Popiołek et al. (2011)
		2012	154	1.9	F	Karamon et al. (2014)
		n.a.	55	0	N	Karamon et al. (2014)
Spain	Madrid	2021	72	0	N	Sanjuán et al. (2022)

P = prevalence

F = fecal analysis

N = necropsy

T = treatment

n.a. = not available

**Table 3:** Occurrence of *Baylisascaris procyonis* in other animal species in Europe

Region	Year of Publication	Investigation period	n	P[%]	Species	Study Method	Reference
**France**							
Savoyen (Auvergne-Rhône-Alpes)	2020	2011–2016	1	Pos.	*Canis lupus* (wolf)	F	Umhang et al. (2020)
**Germany**							
South (n.a.)	1981	1979	65	100	*Myocastor coypus* (nutria)	N	Koch and Rapp (1981)
Soest (North Rhine-Westphalia)	2009	n.a.	2	100	*Amazona aestiva* (blue-fronted amazon)	N	Hillmers and Peters (2009)
Leipzig (City)	2024	n.a.	1	Pos.	*Trichoglossus moluccanus* (rainbow lorikeet)	N	Pfetzing et al. (2024)
**Ireland**							
Dublin (City)	1966	n.a.	n.a.	Pos.	*Castor canadensis* (beaver)	N	Kelly and Innes (1966)
**Spain**							
Lugo (City)	2015	2013	2	100	*Eulemur albifrons* (white-headed lemur)	N + T	Jimenez Martinez et al. (2015)

n = sample size

P = prevalence

Pos*. =* positive result in a single animal or without prevalence given

n.a. = not available

F = fecal analysis

N = necropsy

T = treatment

Between 1990 and 2004, studies were conducted in Hesse, Saxony-Anhalt, and Brandenburg (Fig. 1). *Baylisascaris procyonis* was detected with a prevalence of 71.2–80% in Hesse and 39.3% in Saxony-Anhalt (Bauer et al. 1992; Gey 1998; Hohmann et al. 2002; Winter et al. 2005). No infestation was detected in prevalence studies in Brandenburg during this period, but evidence was found in a single raccoon (Lux and Priemer 1995a, b; Schwarz et al. 2015).

**Fig. 2:**
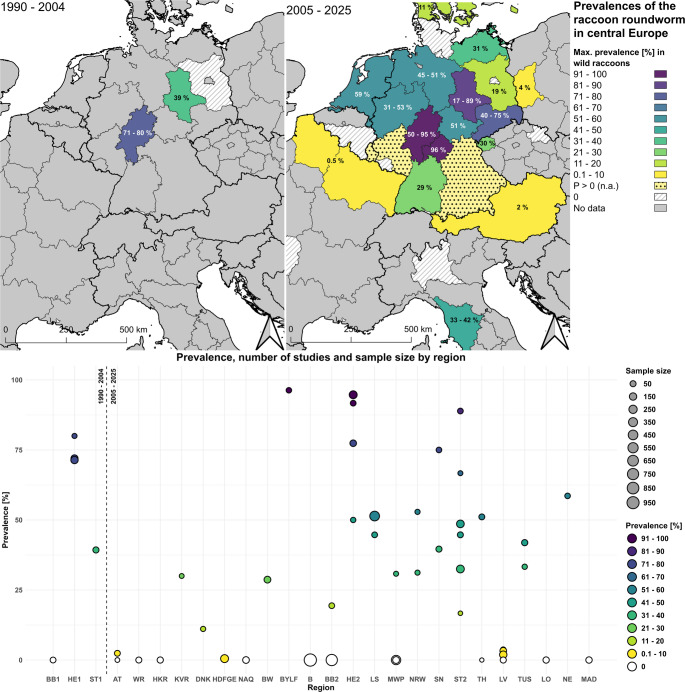
Prevalence ranges of *Baylisascaris procyonis* in free ranging raccoons in central Europe at NUTS level 0, 1 or 2 for 1990–2004 (left) and 2005–2025 (right). The prevalences and sample sizes of each study (Table 2) are shown for each region (bottom) (AT = Austria; Belgium: WR = Walloon Region; Czech Republic: HKR = Hradec Kralove Region, KVR = Karlovy Vary Region; DNK = Denmark; France: HDFGE = Hauts-de-France & Grand Est, NAQ = Nouvelle-Aquitaine; Germany: BW = Baden-Württemberg, BYLW = Bayern (Lower Franconia), B = Berlin, BB = Brandenburg, HE = Hesse, LS = Lower Saxony, MWP = Mecklenburg-Western Pomerania, NRW = North Rhine-Westphalia, SN = Saxony, ST = Saxony-Anhalt, TH = Thuringia; Poland: LV = Lubusz Voivodeship; Italy: TUS = Tuscany, LO = Lombardy; NE = Netherlands; Spain: MAD = Madrid). Studies without sample size are indicated as positive (P > 0 (n.a.)) or negative (P = 0%) and are listed in Table S1. n.a. = not available

Between 2005 and 2025, *B. procyonis* was detected in wild raccoons in 11 of Germany’s 16 federal states (e.g. Baden-Württemberg (Reinhardt et al. 2023); Bavaria (Heddergott et al. 2020; Peter et al. 2023); Brandenburg (Peter et al. 2024); Hesse (Peter et al. 2023); Lower Saxony (Anheyer-Behmenburg 2013); Mecklenburg-Western Pomerania (Rentería-Solís et al. 2025); North Rhine-Westphalia (Rentería-Solís et al. 2025); Rhineland-Palatinate (Heddergott et al. 2020); Saxony (Rentería-Solís et al. 2018); Saxony-Anhalt (Peter et al. 2024); Thuringia (Rentería-Solís et al. 2020)). A detailed map of prevalence rates in central Europe is shown in Fig. 2. In Germany, the prevalence rates range from 19.4% in Brandenburg to 96.3% Bavaria (Lower Franconia). In other European countries, the rates range from 0.5% in France to 58.6% in the Netherlands. With the exception of Italy, all cases of *B. procyonis* in wild raccoons were detected in countries bordering Germany (Popiołek et al. 2011; Al-Sabi et al. 2015; Duscher et al. 2021; Maas et al. 2022; Frantz et al. 2024; Umhang et al. 2024; Benovics et al. 2025). In most cases, the evidence was found i areas close to the German border (Popiołek et al. 2011; Duscher et al. 2021; Maas et al. 2022; Frantz et al. 2024; Umhang et al. 2024; Benovics et al. 2025). To date, no *B. procyonis* infections have been etected in wild racoons in studies in Belgium and Spain (Maas et al. 2022; Sanjuán et al. 2022).

The raccoon roundworms from raccoon populations in central Germany, the Netherlands, Belgium, Luxembourg, and northern France were genetically analyzed. They show at least three haplotypes for the species *B. procyonis* in Europe (Osten-Sacken et al. 2018; Maas et al. 2022; Heddergott et al. 2023; Frantz et al. 2024; Umhang et al. 2024). Two of these three clusters are concentrated in central Germany, while the third cluster has been identified in the border region between France, Belgium, and Luxembourg (Osten-Sacken et al. 2018; Frantz et al. 2024; Umhang et al. 2024).

**Fig. 3:**
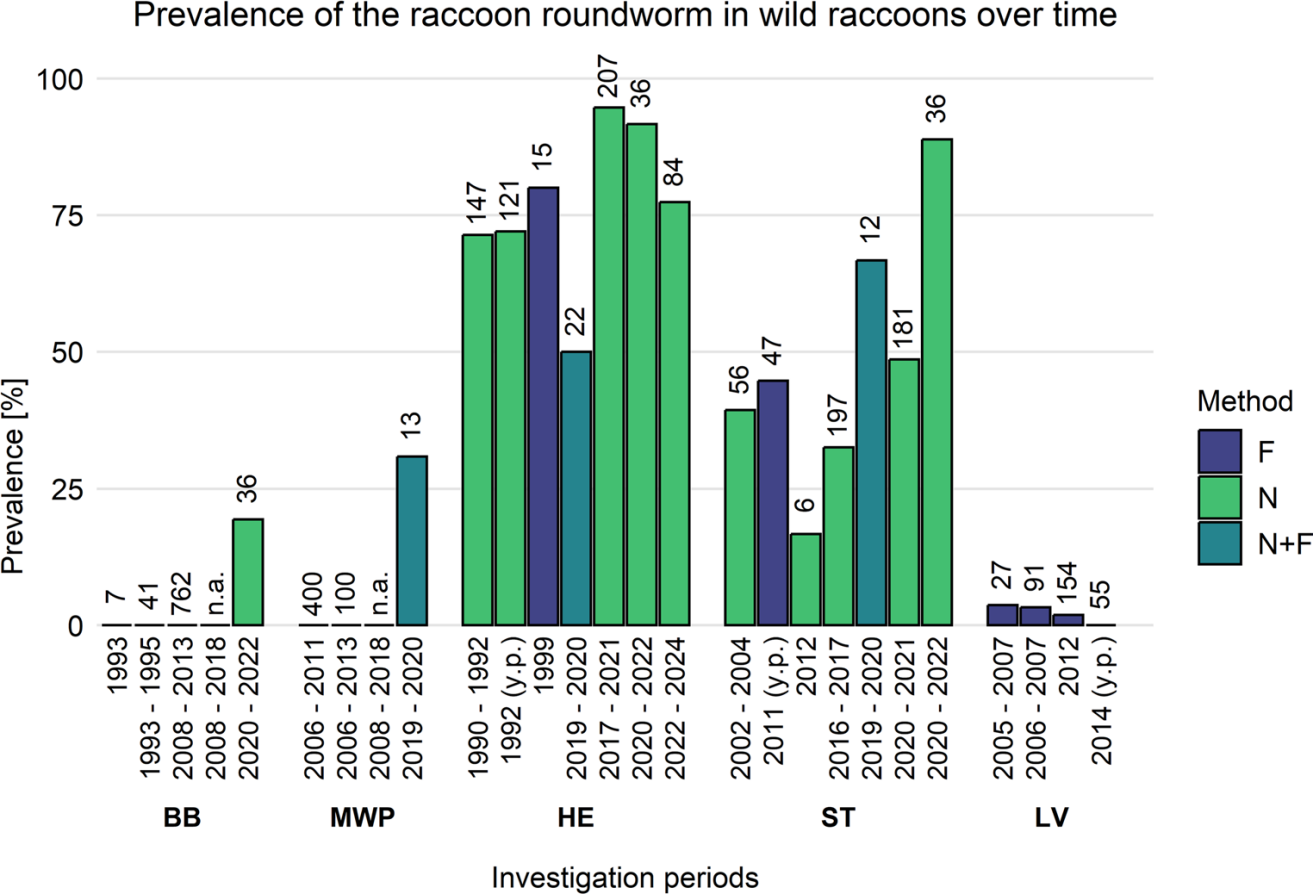
Temporal development of *Baylisascaris procyonis* prevalence (P [%]) in wild raccoons. The study period (bottom) and the sample size (top) are indicated. If the study period was not available, the year of publication (y.p.) was used. Germany: BB = Brandenburg, MWP = Mecklenburg-Western Pomerania, HE = Hesse, ST = Saxony-Anhalt; Poland: LV = Lubusz Voivodeship. Colors indicate the study method used: F = fecal analysis, N = necropsy, N + F = necropsy and fecal analysis. In Brandenburg only necropsies were carried out, in Lubusz only fecal analysis. In the first study from Mecklenburg-Western Pomerania fecal analysis was carried out, in the following two studies necropsy

At five locations, several studies (> 3) were conducted on the *B. procyonis* prevalence in wild raccoons. These are four federal states in Germany (Brandenburg, Mecklenburg-Western Pomerania, Hesse, Saxony-Anhalt) and Lubusz (Poland) (Bauer et al. 1992; Lux and Priemer 1995a, b; Gey 1998; Hohmann et al. 2002; Winter et al. 2005; Bartoszewicz et al. 2008; Helbig 2011; Popiołek et al. 2011; Karamon et al. 2014; Rentería-Solís 2015; Schwarz et al. 2015; Michler 2017; Heddergott et al. 2020, 2023; Peter et al. 2023, 2024; House 2024; Rentería-Solís et al. 2025). For these locations, the development of prevalence over time, specifying the examination method, is shown in Fig. 3. In Brandenburg no evidence of *B. procyonis* was found in between 1993 and 2018 (Lux and Priemer 1995a, b; Schwarz et al. 2015; Heddergott et al. 2020). Newer data from 2020 to 2022 provided evidence for *B. procyonis* with a prevalence of 19.4% (Peter et al. 2024). In Mecklenburg-Western Pomerania no evidence of *B. procyonis* was found in between 2006 and 2018 (Rentería-Solís 2015; Michler 2017; Heddergott et al. 2020). Newer data from 2019 to 2020 provided evidence for *B. procyonis* with a prevalence of 30.8% (Rentería-Solís et al. 2025). In Hesse, prevalence rates varied between 50% and 94.7% between 1990 and 2024 (Bauer et al. 1992; Gey 1998; Hohmann et al. 2002; Peter et al. 2023, 2024; Rentería-Solís et al. 2025). In Saxony-Anhalt, prevalence rates between 2002 and 2022 ranged from 16.7% to 88.9% (Winter et al. 2005; Helbig 2011; Schwarz et al. 2015; Heddergott et al. 2023; House 2024; Peter et al. 2024; Rentería-Solís et al. 2025). In Lubusz, prevalence rates fell from 3.7% to 0% between 2005 and 2014(Bartoszewicz et al. 2008; Popiołek et al. 2011; Karamon et al. 2014). In Brandenburg, only necropsies were performed, in Mecklenburg-Western Pomerania, Hesse and Saxony-Anhalt both necropsies and fecal analyses were performed, and in Lubusz only fecal analyses were performed (Bauer et al. 1992; Lux and Priemer 1995a, b; Gey 1998; Hohmann et al. 2002; Winter et al. 2005; Bartoszewicz et al. 2008; Helbig 2011; Popiołek et al. 2011; Karamon et al. 2014; Schwarz et al. 2015; Heddergott et al. 2023; Peter et al. 2023, 2024; House 2024).

In Germany and Denmark, all published studies on parasitism in raccoons kept in captivity have found infections with *B. procyonis* (Roth 1968; Tscherner 1974; Brinch 2006; Brandes 2007; Davidson et al. 2013; Al-Sabi et al. 2015). The reported prevalences range between 21.1% and 100%.

### In other animals and humans

In Europe, five animal species have been identified as paratenic/accidental hosts to date (Table 3). In Germany, Spain, and Ireland, infections in paratenic/accidental hosts have been detected in zoos and animal farms (Fig. 1). Two bird species (*Trichoglossus moluccanus*, *Amazona aestiva*), one primate species (*Eulemur albifrons*), and two rodent species (*Myocastor copyus*, *Castor canadensis*) were infected with *B. procyonis* (Kelly and Innes 1966; Koch and Rapp 1981; Hillmers and Peters 2009; Jimenez Martinez et al. 2015; Pfetzing et al. 2024). Additionally, genetic evidence of *B. procyonis* was found in wolf feces (*Canis lupus)* in eastern France (Umhang et al. 2020).

There is little information available on the incidence of infection in humans in Europe. In a study conducted in Germany, 4 out of 31 subjects (P = 12.9%) tested positive for antibodies to *B. procyonis* (Conraths et al. 1996). The subjects were assigned to different risk groups at the start of the study; all 4 subjects who tested positive belonged to the 13 people in risk group 1, who had close contact with raccoons (P = 30.8%). In Austria and Gemany, one case of ocular larva migrans was confirmed in each country (Schrott 1961; Küchle et al. 1993; Kazacos 2016). In Germany, another case of ocular larva migrans is suspected to have been caused by *B. procyonis* (Nguyen-Höhl et al. 2021; Weese and Stull 2025).

## Discussion

### *Baylisascaris procyonis* - prevalence in central and western Germany

The results of this study expand the knowledge of the distribution of the abundant nematode species *B. procyonis* in Europe. High prevalences of over 75% for *B. procyonis* are already known for Hesse and indicate continuous infection in raccoon populations and the environment (Gey 1998; Peter et al. 2023, 2024). For Thuringia, data on the prevalence of *B. procyonis* in raccoons was provided for the first time; qualitative evidence had already been documented previously (Heddergott et al. 2020; Rentería-Solís et al. 2020). The districts studied in Thuringia border Hesse (Wartburg district) and Saxony-Anhalt (Sömmerda), which are known hotspots for the spread of *B. procyonis*. However, at 42.9%, the prevalence in the Wartburg district is lower than the prevalence in Hesse (P = 77.4%), whil Sömmerda shows a similar prevalence (P = 80.0%) to Saxony-Anhalt (P = 88.9%) (Heddergott et al. 2023; Peter et al. 2023, 2024). For Nort Rhine-Westphalia, the new data from the Aachen distric show a higher prevalence tha the 31.2% from a previous study in the northeastern part of the state (Rentería-Solís et al. 2025). At 52.9%, the prevalence in the Aachen district is similar to that in the neighboring Netherland (P = 58.6%) (Maas et al. 2022).

### Distribution and spread in Europe

The available prevalence data on raccoons and paratenic/accidental hosts in Europe document a wider spatial distribution and spread of the nematode species (Fig. 1). The center of distribution is in Central Europe, where *B. procyonis* has been established for decades (Figs. 1 and 2). Another distribution point has been added in recent years in Tuscany (Italy) (Fig. 2).

**Fig. 4:**
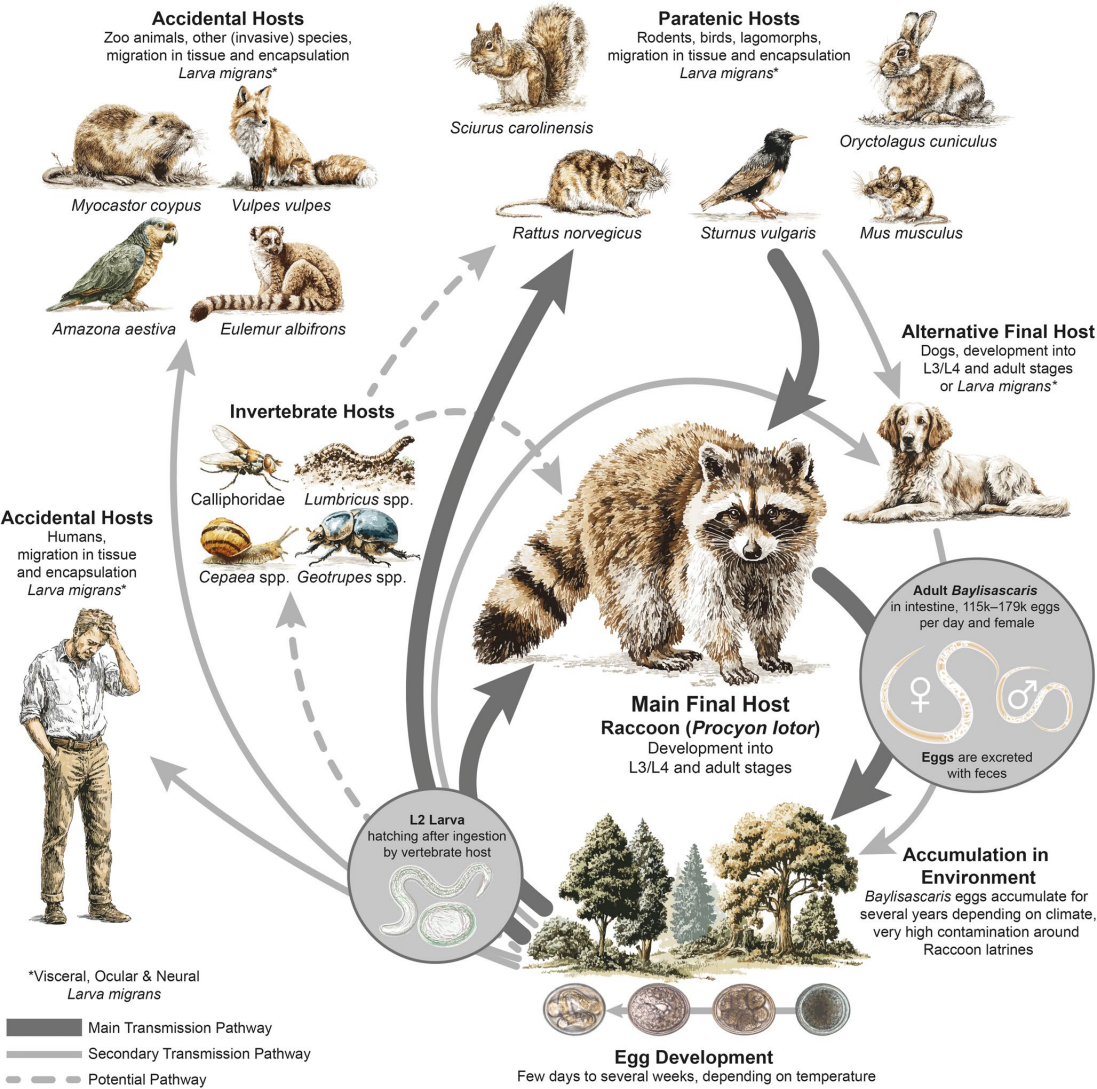
Life cycle of *Baylisascaris procyonis* with focus on the main transmission cycle and secondary transmission pathways. Main transmission cycle involving main host (Raccoon), adult nematodes in small intestine of main host, egg development in the environment and facultative (optional) paratenic hosts. Secondary transmission pathways involving accidental hosts (e.g. humans) and alternative final host (dogs). Also shown: Larvae hatching after ingestion by final/paratenic/accidental host as well as experimentally demonstrated additional pathway involving invertebrates

As *B. procyonis* is primarily dependent on its final host (Fig. 4), its distribution is linked to that of the raccoon (Kazacos 2001; Peter et al. 2023; Stutz et al. 2024). Several haplotypes are known for raccoons in Europe, some of which extend beyond national borders: From Central Europe, at least eleven populations can be genetically differentiated in northern France, Luxembourg, Belgium, the Netherlands, Germany, Poland, the Czech Republic and Austria, which overlap in their distribution areas and interbreed (Frantz et al. 2013, 2021, 2023, 2024; Biedrzycka et al. 2014, 2020a; Fischer et al. 2015, 2017; Osten-Sacken et al. 2018; Heddergott et al. 2020, 2023; Maas et al. 2022; Umhang et al. 2024). In addition, there are two in central/western France, two in Italy, and four in Spain, which are currently isolated from each other but are likely to encounter and intersect with each other in the foreseeable future as they continue to spread (Alda et al. 2013; Fischer et al. 2017; Maillard et al. 2020; Frantz et al. 2021; Larroque et al. 2023; Garofalo et al. 2024). The raccoon populations in northern Italy and Tuscany appear to belong to two different haplotypes which are both prevalent in Central Europe and among the most common in North America (Garofalo et al. 2024). It still needs to be investigated whether these populations and their nematodes originated from new introductions from North America or from existing populations in Central Europe. The population in Tuscany (central Italy) already carries *B. procyonis* and could thus introduce the nematode species into the population in the north of the country (Romeo et al. 2021; Lombardo et al. 2022; Garofalo et al. 2024).

Compared to the natural range of the raccoon roundworm in North America, its genetic diversity in Europe is significantly reduced due to the founder effect (Osten-Sacken et al. 2018; Carlson et al. 2021; Frantz et al. 2024). Two of the three identified genetic clusters of the raccoon roundworm originate from the “Hesse” (western cluster) and “Harz” (eastern cluster) raccoon populations in central Germany (Osten-Sacken et al. 2018; Frantz et al. 2024; Umhang et al. 2024). These two populations are among the oldest in Europe, and their founder animals must have already been infected when they were released (Gey 1998; Frantz et al. 2021; Heddergott et al. 2023). Raccoon roundworms originating from the “western cluster” have also been detected in local raccoon populations in Austria, the Netherlands, and Luxembourg (Duscher et al. 2021; Frantz et al. 2024; Umhang et al. 2024). The third cluster, located in the border region between France, Belgium, and Luxembourg, was identified in both local raccoon populations and animals of mixed origin. The origins included the “Hesse” raccoon population, which has spread further westward (Frantz et al. 2024). It is possible that the raccoon population in Tuscany has introduced a fourth roundworm haplotype into Europe. Genetic testing is needed to determine the overlap with existing haplotypes.

The spread of the roundworm can also be observed in the east. In areas/populations such as Brandenburg, Mecklenburg-Western Pomerania, and parts of Saxony-Anhalt, which were long considered “roundworm-free,” recent studies have shown the presence of *B. procyonis* (Heddergott et al. 2023; Peter et al. 2024; Rentería-Solís et al. 2025). The latest studies detected positive prevalences in northwestern Brandenburg and southeastern Mecklenburg-Western Pomerania, while no evidence of infestation in these regions was found in several previous investigations spanning from 2006/2008 to 2018 (Rentería-Solís 2015; Schwarz et al. 2015; Michler 2017; Heddergott et al. 2020; Peter et al. 2024; Rentería-Solís et al. 2025). In north-western Brandenburg, it is highly likely that infected raccoons migrated from neighboring Saxony-Anhalt and introduced the roundworm in the new area, but to accurately determine the introduction history in Brandenburg and Mecklenburg-Western Pomerania genetic testing is required. Research using genetic methods has confirmed that migrating raccoons have introduced roundworms to new areas in Luxembourg, Austria, and Saxony-Anhalt (Duscher et al. 2021; Heddergott et al. 2023; Frantz et al. 2024). Furthermore, the introduction of roundworms into new populations and areas can be facilitated by the release or escape of infected raccoons from animal husbandry facilities (Frantz et al. 2024).

In Norway, six raccoons were to be confiscated from a farm in the south. Two managed to escape beforehand, and the four remaining animals were examined by necropsy (Davidson et al. 2013). Given the high infestation levels of these animals (mA = 53, P = 100%), it cannot be discounted that the escaped animals were also infested and had the potential to disseminate the roundworm in the wild. There is no available data on infections in raccoons for the French Alpine region, which lies outside the known French raccoon populations in northern, western, and central France (Maillard et al. 2020; Larroque et al. 2023; Umhang et al. 2024). However, the detection of *B. procyonis* in wolf feces (Umhang et al. 2020) suggests the presence of an infection within a raccoon population inhabiting the same environment. This finding indicates that the distribution of *B. procyonis* may extend beyond previously recognized limits. In consideration of the potential dispersal of raccoons throughout Europe, there are significant regions that are suitable but have not yet been colonized, as well as areas that are becoming increasingly suitable as a consequence of climate change (Kochmann et al. 2021; Cunze et al. 2023). Since the raccoon roundworm is linked to the spread of raccoons, it also has great potential for spreading throughout Europe. In this regard, the spread of the raccoon roundworm does not appear to be hindered by climatic factors due to the temperate conditions in Europe (Stutz et al. 2024).

Data on the occurrence of *B. procyonis* in Europe was collected using four methods of investigation: necropsy, flotation of fecal samples, DNA detection in fecal samples, and visualization of adult nematodes in feces after administration of antihelminthic drugs. Of these methods, necropsy is considered the most reliable. However, it is both costly and time-consuming (Page et al. 2005, 2025). Compared with necropsy, fecal analysis by flotation is significantly less sensitive. Eggs may be misidentified as other species of Ascarididae when examining environmental samples, as morphological differentiation can be difficult under certain conditions (Kazacos and Turek 1983; Zajac and Conboy 2012). Nevertheless, it is a viable option for examining large sample quantities and assessing environmental contamination at latrine sites (Page et al. 2005, 2025). However, conclusions about prevalence based on raccoon fecal analyses may significantly underestimate the actual percentage, as infections with nematodes that are not yet potent may be overlooked (Gey 1998; Lombardo et al. 2023; Reinhardt et al. 2023; Page et al. 2025). Although genetic tests are considered to be very sensitive, they require a certain number of eggs in the feces to be effective. Therefore, they are not suitable for lower prevalence/intensity levels (Page et al. 2025). As examination of the feces is necessary, medication against helminths can only be used to detect parasites in captive raccoons (Tenora and Staněk 1990; Maas et al. 2022; Frantz et al. 2024).

In addition to the research method used, different sample sizes make it difficult to directly compare individual prevalence data. Given the expected prevalence, samples of fewer than 15 animals are usually inadequate. (Sarabeev et al. 2025). Sample sizes of 15 or more are only suitable for expected prevalence rates between 10% and 90% (Sarabeev et al. 2025). Sample szes of oer 100 are recommended for prevalence rates that are either very high or very low. The prevalence of raccoon infestation is also influenced by demographics and seasonality. In their natural habitat, juvenile raccoons often exhibit higher infestation rates than adult animals (Snyder and Fitzgerald 1985; Kazacos 2001; Page et al. 2009; Yeitz et al. 2009; French et al. 2019). This has also been observed in some European studies, but no significant differences were found in most cases (Bauer et al. 1992; Gey 1998; Anheyer-Behmenburg 2013; Rentería-Solís et al. 2018; Biedrzycka et al. 2020b; Maas et al. 2022; Lombardo et al. 2023; Reinhardt et al. 2023). This could be due to smaller sample sizes or significant differences in the age distribution of the sample. The infection rate is also affected by the time of investigation: higher or lower prevalence rates may be observed depending on the season (Page et al. 2009, 2016; French et al. 2019). These fluctuations are attributed to the poorer conditions for adult nematodes in winter, caused by a reduced food supply and reduced body mass, as well as the associated “self-healing” of raccoons (Kazacos 2001; Page et al. 2009, 2016). This effect has not been observed in Europe, but seasonality has been little studied to date (Gey 1998; Lombardo et al. 2023).

For these reasons, the actual prevalence rates in Europe may be considerably higher or lower than the reported data suggests. Hesse and Saxony-Anhalt (Germany), the regions with the largest number of studies on *B. procyonis* in Europe, show considerable fluctuations and no clear trend in the prevalence data (Fig. 3). This may be due to differences in sample sizes, testing methods and testing periods. In Lubusz (Poland), the drop in prevalence to 0% in the latest study is also likely to be due to the sample size and survey method rather than the nematode having disappeared from this area. Clear trends in prevalence within a population or area can only be identified through continuous surveys using sensitive methods and sufficient sample sizes.

### Paratenic hosts and infections in humans

*Baylisascaris procyonis* can infect several animal species and humans as paratenic/accidental hosts and cause larva migrans. The estimated incubation period is 1–4 weeks (Kazacos 2001; Graeff-Teixeira et al. 2016). Although it is known that more than 150 species can act as paratenic hosts (Graeff-Teixeira et al. 2016), there is no available data from the wild in Europe. The cases of larva migrans in paratenic/accidental hosts in Europe have been documented in five non-native species that were exclusively kept in zoos or animal husbandry facilities (Kelly and Innes 1966; Koch and Rapp 1981; Hillmers and Peters 2009; Jimenez Martinez et al. 2015; Pfetzing et al. 2024). Of these species, the nutria (*Myocastor coypus*) is the only one that can be found in the wild in Europe. In an experimental study conducted in North America, 94% of common starlings (*Sturnus vulgaris*) fed on *B. procyonis*-infected earthworms (*Lumbricus terrestris*) showed typical symptoms of larva migrans, 61% died (Henke 2025). European starlings and *L. terrestis* are considered invasive species in North America, yet they are among the most widespread native bird and earthworm species in Europe (Gailing et al. 2012; Heldbjerg et al. 2019; Henke 2025). In additio to ingesting eggs directly from the environment, earthworms seem to act as an alternate route of infection for paratenic hosts (Henke 2025). This could represent an additional optional step in the life cycle of *B. procyonis* (Fig. 4). In addition to earthworms, other invertebrates, such as flies, snails and beetles, may also act as a pathway for the transmission of *B. procyonis* for either paratenic hosts or its final host. Raccoons in Germany have been shown to use the common starling, annelids, gastropods and several insect species as food sources (Peter et al. 2024).

Keeping raccoons and potential paratenic hosts simultaneously or successively, or having contact with free-roaming raccoons via outdoor enclosures, always poses a certain risk of infection to the paratenic hosts. The nematodes found in the Canadian beavers in Dublin were not identified, and there is no information about possible contact with raccoons. However, based on the symptoms and morphology, it was most likely *B. procyonis* (Kelly and Innes 1966; Kazacos 2001). In two cases, one in Spain and one in Germany, the affected animals had either been in contact with raccoons from the same facility or had possibly come into contact with wild raccoons that had been seen outside the outdoor enclosure (Jimenez Martinez et al. 2015; Pfetzing et al. 2024). In two other cases in Germany, raccoons had previously been kept in the same enclosure, which was then cleaned and disinfected, but without success (Koch and Rapp 1981; Hillmers and Peters 2009). Some captive raccoons were found to have very high prevalence rates (Brandes 2007; Davidson et al. 2013), and the infectious eggs are difficult to eliminate from enclosures due to their resistance to common disinfectants (Gavin et al. 2005; Kazacos 2016). Although, in addition to raccoons, dogs can also serve as final hosts for *B. procyonis* (although less successfully) (Sapp et al. 2020), no infection has been detected in a wolf to date. Therefore, the genetic evidence in wolf feces (Umhang et al. 2020) could be explained by the pathogen passing through the wolf’s intestines after it ingested eggs from the environment or consumed an infected paratenic host/raccoon.

Although there is no data on the infectious dose for humans, it is assumed to be less than 5,000 eggs (Sorvillo et al. 2002). This is equivalent to the average egg output of a female raccoon nematode in about 300 mg of raccoon feces (Reed et al. 2012). However, the actual infectious dose could be significantly lower, based on related nematode species (Sorvillo et al. 2002; Wise et al. 2005). While most human infections are asymptomatic, the symptoms and severity of the disease depend on the number of eggs ingested and the location of the larvae in the body. People who come into close contact with raccoons through work or other activities are at an increased risk of coming into contact with raccoon roundworm and developing antibodies. Results from Germany and North America show that 100% and 79.2% of seropositive subjects, respectively, reported contact with raccoons (Conraths et al. 1996; Sapp et al. 2016). There are no large-scale studies on the incidence of infection in the European population. Antibodies were detected in 7% of California adults and 7% of Chicago children, despite no documented contact with raccoons (Brinkman et al. 2003; Weinstein et al. 2017). In addition to cases in the endemic region of North America, infections in Germany and Austria confirm the presence of this disease in Europe (Schrott 1961; Küchle et al. 1993; Nguyen-Höhl et al. 2021). All three cases in Europe resulted in permanent visual impairment. Children under the age of four and people with behavioral disorders such as pica or geophagy are generally at an increased risk of ingesting an infectious dose of eggs (Kazacos 2001; Weese and Stull 2025). Of the 60 confirmed cases, 25% involved individuals observed eating soil, feces, or other potentially contaminated materials (Weese and Stull 2025). Studies from the UK, Lithuania, Switzerland and Germany found that 3.08% to 4.98% of children aged 3 to 14 exhibited pica behavior, while 1.1% of adults exhibited pica behavior (Hartmann et al. 2018, 2022; Murray et al. 2018; Lesinskienė et al. 2023; Papini et al. 2024). Children under the age of four naturally go through a phase where they frequently put their hands in their mouths. During this phase, they are thought to ingest between 10 and 1,000 mg of soil and dust per day, depending on the estimation method used (Moya and Phillips 2014).

Due to the high local prevalence and increasing population densities of the final hosts, as well as the increased contact frequency resulting from urbanization, high-risk human behavior (e.g. contact with raccoons, pica or geophagy) and the lack of diagnostic methods, it can be assumed that there is an unknown number of unreported cases of Baylisascariasis in areas where *B. procyonis* is prevalent in Europe.

## Conclusion

The available data show a wide distribution of *B. procyonis* in Central Europe, with locally high prevalences. Genetic studies confirm several points of spread and an expansion of the range of the parasite. However, large parts of Europe with raccoon populations remain unexplored and, in some areas, only less sensitive fecal analyses have been carried out. This means that *B. procyonis* could already be more widespread than previously assumed. Due to increasing population densities and additional uncontrolled releases of raccoons, further spread across Europe is highly likely. Apart from a few documented cases, there is currently a lack of valid information on the prevalence of infection in paratenic hosts and humans in Europe. Therefore, extensive studies are necessary to fill the gaps in our knowledge regarding the incidence of infection in the population, the actual spread and spread patterns, as well as the life cycle of this species in Europe. This is also necessary to provide the public with comprehensive information about the possible dangers of Baylisascariasis. Additionally, the feasibility of establishing a European test center for Baylisascariasis in humans should be examined.

## Supplementary Information

Below is the link to the electronic supplementary material.

Supplementary Material 1 Table S1 Occurrence data on Baylisascaris procyonis in wild and captive raccoons in Europe (DOCX 239 KB)

## Data Availability

The raw data on which the calculations are based and which support the conclusions of this article will be made available by the authors without reservation on request.
