# Supplementary material for: Baylisascaris procyonis on the rise in Europe: a comprehensive review and analysis of occurrence data
Source: Parasitol Res. 2025 Dec 9;124(12):157. doi: 10.1007/s00436-025-08611-z (PMC12689684; doi:10.1007/s00436-025-08611-z)
Supplement: Supplementary file 1 — Supplementary Material 1 Table S1 Occurrence data on Baylisascaris procyonis in wild and captive raccoons in Europe (DOCX 239 KB) [file 436_2025_8611_MOESM1_ESM.docx]

| **Region** | **Year of publication** | **Investigation period [years]** | **n** | **P [%]** | **Study Methods** | **In captivity** | **Notes** | **Reference** |
| --- | --- | --- | --- | --- | --- | --- | --- | --- |
| **Austria** |  |  |  |  |  |  |  |  |
| Salzburg, Lower Austria, Styria, Carinthia | 2017 | 2010 - 2016 | 8 | 0 | N |  |  | Duscher et al. (2017) |
| Vorarlberg, Upper Austria, Lower Austria, Styria, Burgenland, Salzburg | 2021 | 2017 - 2019 | 41 | 0 | N |  |  | Duscher et al. (2021) |
| Vorarlberg | 2021 | 2019 | 1 | Pos. | N |  |  | Duscher et al. (2021) |
| **Belgium** |  |  |  |  |  |  |  |  |
| Walloon Region | 2022 | 2012 - 2015 | 50 | 0 | N |  |  | Maas et al. (2022) |
| Walloon Region | 2024 | 2023 | 1 | in. | T |  |  | Frantz et al. (2024) |
| **Czech Republic** |  |  |  |  |  |  |  |  |
| Brno (City) | 1990 | n.a. | 1 | Pos. | T | X |  | Tenora and Staněk (1990) |
| Hradec Kralove Region | 2020 | 2012 - 2017 | 62 | 0 | N |  |  | Biedrzycka et al. (2020) |
| Karlovy Vary Region | 2025 | 2023 - 2025 | 10 | 30 | N |  |  | Benovics et al. (2025) |
| **Denmark** |  |  |  |  |  |  |  |  |
| / | 2006 | n.a. | 19 | 21.1 | F | X |  | Brinch (2006) |
| / | 2015 | 2009 - 2015 | 3 | 33.3 | F | X | n = Zoos | Al-Sabi et al. (2015) |
| / | 2015 | 2009 - 2015 | 18 | 11.1 | N |  |  | Al-Sabi et al. (2015) |
| **France** |  |  |  |  |  |  |  |  |
| Hauts-de-France & Grand-Est | 2024 | 2011 - 2021 | 207 | 0 | N |  |  | Umhang et al. (2024) |
| Grand-Est | 2024 | 2021 | 1 | Pos. | N |  |  | Umhang et al. (2024) |
| Nouvelle-Aquitaine | 2024 | 2019 - 2022 | 92 | 0 | N |  |  | Umhang et al. (2024) |
| **Germany** |  |  |  |  |  |  |  |  |
| / | 1968 | 1952 – 1961 | 7 | 28.6 | F | X |  | Roth (1968) |
|  | 2007 | 2003 | 16 | 37.5 | F | X |  | Brandes (2007) |
| Center | 2020 | 2008 - 2018 | 8158 | ~ 43.6 | N |  | several states combined | Heddergott et al. (2020) |
| Baden-Württemberg (Widdern) | 1956 | 1950 | 1 | Pos. | F+T | X |  | Sprehn and Haakh (1956) |
| Baden-Württemberg | 2020 | 2008 - 2018 | n.a. | Pos. | N |  |  | Heddergott et al. (2020) |
|  | 2025 | 2019 - 2020 | 1 | Neg. | N+F |  |  | Rentería-Solís et al. (2025) |
|  | 2023 | 2019 - 2020 | 101 | 28.7 | N |  |  | Reinhardt et al. (2023) |
|  | 2023 | 2019 - 2020 | 101 | 9.9 | F |  | same n as above, but different method | Reinhardt et al. (2023) |
| Bavaria | 2020 | 2008 - 2018 | n.a. | Pos. | N |  |  | Heddergott et al. (2020) |
| Bavaria (Lower Franconia) | 2023 | 2017 - 2021 | 27 | 96.3 | N |  |  | Peter et al. (2023) |
| Berlin | 1974 | 1970 – 1973 | n.a. | Pos. | F | X |  | Tscherner (1974) |
|  | 2015 | 2006 - 2013 | 140 | 0 | N |  |  | Rentería-Solís (2015) |
|  | 2020 | 2008 - 2018 | n.a. | 0 | N |  |  | Heddergott et al. (2020) |
| Brandenburg (Neuruppin) | n.a. | 1995 | 1 | Pos. | n.a. |  | unpub. data, oral com., no method given | Schwarz et al. (2015) |
| Brandenburg | 1995 | 1993 | 7 | 0 | N |  |  | Lux and Priemer (1995a) |
|  | 1995 | 1993 - 1995 | 41 | 0 | N |  |  | Lux and Priemer (1995b) |
|  | 2015 | 2008 - 2013 | 762 | 0 | N |  |  | Schwarz et al. (2015) |
|  | 2020 | 2008 - 2018 | n.a. | 0 | N |  |  | Heddergott et al. (2020) |
|  | 2025 | 2019 - 2020 | 1 | Neg. | N+F |  |  | Rentería-Solís et al. (2025) |
|  | 2024 | 2020 - 2022 | 36 | 19.4 | N |  |  | Peter et al. (2024) |
| Hamburg | 2020 | 2008 - 2018 | n.a. | 0 | N |  |  | Heddergott et al. (2020) |
| Hesse | 1992 | n.a. | 121 | 72 | N |  |  | Bauer et al. (1992) |
|  | 1998 | 1990 - 1992 | 147 | 71.4 | N |  |  | Gey (1998) |
|  | 1998 | 1990 - 1992 | 138 | 54.3 | F |  | same animals as above, but different method | Gey (1998) |
|  | 1998 | 1990 - 1992 | 38 | 68.4 | N |  | wild animals kept in an enclosure for a short time | Gey (1998) |
|  | 1998 | 1990 - 1992 | 38 | 81.6 | F |  | same n as above, but different method | Gey (1998) |
|  | 2002 | 1999 | 15 | 80 | F |  |  | Hohmann et al. (2002) |
|  | 2018 | 2011 - 2015 | n.a. | Pos. | N |  |  | Osten-Sacken et al. (2018) |
|  | 2020 | 2008 - 2018 | n.a. | Pos. | N |  |  | Heddergott et al. (2020) |
|  | 2025 | 2019 - 2020 | 22 | 50 | N+F |  |  | Rentería-Solís et al. (2025) |
|  | 2023 | 2017 - 2021 | 207 | 94.7 | N |  |  | Peter et al. (2023) |
|  | 2024 | 2020 - 2022 | 36 | 91.7 | N |  |  | Peter et al. (2024) |
|  | New data | 2022 - 2024 | 84 | 77.4 | N |  |  | New data |
| Lower Saxony (Lüneburg) | 1956 | 1952 | 2 | Pos. | N | X |  | Sprehn and Haakh (1956) |
| Lower Saxony | 2013 | 2011 - 2013 | 457 | 51.4 | N |  |  | Anheyer-Behmenburg (2013) |
|  | 2013 | 2011 - 2013 | 222 | 5.9 | F |  | same animals as above (negative in necropsy), different method | Anheyer-Behmenburg (2013) |
|  | 2018 | 2011 - 2015 | n.a. | Pos. | N |  |  | Osten-Sacken et al. (2018) |
|  | 2020 | 2008 - 2018 | n.a. | Pos. | N |  |  | Heddergott et al. (2020) |
|  | 2025 | 2019 - 2020 | 38 | 44.7 | N+F |  |  | Rentería-Solís et al. (2025) |
| Mecklenburg-Western Pomerania | 2017 | 2006 - 2011 | 400 | 0 | F |  |  | Michler (2017) |
|  | 2015 | 2006 - 2013 | 100 | 0 | N |  |  | Rentería-Solís (2015) |
|  | 2020 | 2008 - 2018 | n.a. | 0 | N |  |  | Heddergott et al. (2020) |
|  | 2025 | 2019 - 2020 | 13 | 30.8 | N+F |  |  | Rentería-Solís et al. (2025) |
| North Rhine-Westphalia | 2020 | 2008 - 2018 | n.a. | Pos. | N |  |  | Heddergott et al. (2020) |
|  | 2025 | 2019 - 2020 | 16 | 31.2 | N+F |  |  | Rentería-Solís et al. (2025) |
|  | New data | 2022 - 2024 | 17 | 52.9 | N |  |  | New data |
| Rhineland-Palatinate | 2020 | 2008 - 2018 | n.a. | Pos. | N |  |  | Heddergott et al. (2020) |
| Saarland | 2020 | 2008 - 2018 | n.a. | 0 | N |  |  | Heddergott et al. (2020) |
| Saxony | 2020 | 2008 - 2018 | n.a. | Pos. | N |  |  | Heddergott et al. (2020) |
|  | 2018 | 2017 - 2018 | 32 | 75 | N |  |  | Rentería-Solís et al. (2018) |
|  | 2025 | 2019 - 2020 | 53 | 39.6 | N+F |  |  | Rentería-Solís et al. (2025) |
| Saxony-Anhalt | 2005 | 2002 - 2004 | 56 | 39.3 | N |  |  | Winter et al. (2005) |
|  | 2011 | n.a. | 47 | 44.7 | F |  |  | Helbig (2011) |
|  | n.a. | 2012 | 6 | 16.7 | N |  | unpub. data, oral com. | Schwarz et al. (2015) |
|  | 2018 | 2011 - 2015 | n.a. | Pos. | N |  |  | Osten-Sacken et al. (2018) |
|  | 2024 | 2016 - 2017 | 197 | 32.5 | N |  |  | House (2024) |
|  | 2020 | 2008 - 2018 | n.a. | Pos. | N |  |  | Heddergott et al. (2020) |
|  | 2025 | 2019 - 2020 | 12 | 66.7 | N+F |  |  | Rentería-Solís et al. (2025) |
|  | 2023 | 2020 - 2021 | 181 | 48.6 | N |  |  | Heddergott et al. (2023) |
|  | 2024 | 2020 - 2022 | 36 | 88.9 | N |  |  | Peter et al. (2024) |
| Schleswig-Holstein | 2020 | 2008 - 2018 | n.a. | 0 | N |  |  | Heddergott et al. (2020) |
| Thuringia | 2020 | 2008 - 2018 | n.a. | Pos. | N |  |  | Heddergott et al. (2020) |
|  | 2020 | n.a. | 27 | Pos. | n.a. |  |  | Rentería-Solís et al. (2020) |
|  | 2025 | 2019 - 2020 | 5 | 0 | N+F |  |  | Rentería-Solís et al. (2025) |
|  | New data | 2022 - 2024 | 45 | 51.1 | N |  |  | New data |
| **Germany, Poland** |  |  |  |  |  |  |  |  |
| Center, East (G); West (P) | 2020 | 2012 - 2017 | 175 | 4.6 | N |  |  | Biedrzycka et al. (2020) |
| **Italy** |  |  |  |  |  |  |  |  |
| Lombardy | 2021 | 2017 - 2019 | 67 | 0 | N |  |  | Romeo et al. (2021) |
| Tuscany | 2022 | 2021 | 21 | 33.3 | N |  |  | Lombardo et al. (2022) |
|  | 2023 | 2020 - 2022 | 62 | 41.9 | N |  |  | Lombardo et al. (2023) |
|  | 2023 | 2020 - 2022 | 62 | 38.7 | F |  | same n as above, but different method | Lombardo et al. (2023) |
| **Luxembourg** |  |  |  |  |  |  |  |  |
|  | 2020 | 2008 - 2018 | 26 | 0 | N |  |  | Heddergott et al. (2020) |
|  | 2024 | 2023 | 1 | Pos. | N |  |  | Frantz et al. (2024) |
|  | 2024 | 2023 | 6 | ≥ 16.7 | T |  | wild animals kept in an enclosure for a short time | Frantz et al. (2024) |
| **Netherlands** |  |  |  |  |  |  |  |  |
| Drenthe | 2022 | 2019 | 1 | Neg. | F+T |  |  | Maas et al. (2022) |
| Gelderland | 2022 | 2014 | 2 | 100 | N |  |  | Maas et al. (2022) |
| Limburg | 2022 | 2015 - 2019 | 15 | 60 | N+F+T |  |  | Maas et al. (2022) |
|  | 2022 | 2020 | 10 | 60 | F+T |  |  | Maas et al. (2022) |
| North Brabant | 2022 | 2016 | 1 | Neg. | N |  |  | Maas et al. (2022) |
| **Norway** |  |  |  |  |  |  |  |  |
| Southern Norway | 2013 | 2011 | 4 | 100 | N+F | X |  | Davidson et al. (2013) |
| **Poland** |  |  |  |  |  |  |  |  |
| Lodz (City) | 1951 | 1948 | 1 | Pos. | N | X |  | Stefanski and Zarnowski (1951) |
| Lubusz Voivodeship | 2008 | 2005 - 2007 | 27 | 3.7 | F |  |  | Bartoszewicz et al. (2008) |
|  | 2011 | 2006 - 2007 | 91 | 3.3 | F |  |  | Popiołek et al. (2011) |
|  | 2014 | 2012 | 154 | 1.9 | F |  |  | Karamon et al. (2014) |
|  | 2014 | n.a. | 55 | 0 | N |  |  | Karamon et al. (2014) |
|  | 2014 | n.a. | 53 | 0 | F |  | same animals as above, but different method | Karamon et al. (2014) |
| **Spain** |  |  |  |  |  |  |  |  |
| Lugo (City) | 2015 | 2013 | 3 | 0 | N+F | X | Lemurs in same zoo positive (Table 3) | Jimenez Martinez et al. (2015) |
| Madrid | 2022 | 2021 | 72 | 0 | N |  |  | Sanjuán et al. (2022) |

n = sample size

P = prevalence

n.a. = not available

Pos. = positive result in a single raccoon or without prevalence given

Neg. = negative result in a single raccoon

in. = inconclusive

unpub. = unpublished

com. = communication

F = fecal analysis

N = necropsy

T = treatment
